# Fenofibrate, but not ezetimibe, prevents fatty liver disease in mice lacking phosphatidylethanolamine *N*-methyltransferase[S]

**DOI:** 10.1194/jlr.M070631

**Published:** 2017-03-29

**Authors:** Jelske N. van der Veen, Susanne Lingrell, Xia Gao, Abhijit Takawale, Zamaneh Kassiri, Dennis E. Vance, René L. Jacobs

**Affiliations:** Group on the Molecular and Cell Biology of Lipids,* University of Alberta, Edmonton, Alberta, Canada; Department of Biochemistry,† University of Alberta, Edmonton, Alberta, Canada; Department of Physiology,§ University of Alberta, Edmonton, Alberta, Canada; Department of Agricultural, Food, and Nutritional Science,** University of Alberta, Edmonton, Alberta, Canada

**Keywords:** nonalcoholic fatty liver disease, phosphatidylcholine, peroxisome proliferator-activated receptor-α, fibric acids, steatohepatitis, liver fibrosis

## Abstract

Mice lacking phosphatidylethanolamine *N*-methyltransferase (PEMT) are protected from high-fat diet (HFD)-induced obesity and insulin resistance. However, these mice develop severe nonalcoholic fatty liver disease (NAFLD) when fed the HFD, which is mainly due to inadequate secretion of VLDL particles. Our aim was to prevent NAFLD development in mice lacking PEMT. We treated *Pemt^−/−^* mice with either ezetimibe or fenofibrate to see if either could ameliorate liver disease in these mice. Ezetimibe treatment did not reduce fat accumulation in *Pemt^−/−^* livers, nor did it reduce markers for hepatic inflammation or fibrosis. Fenofibrate, conversely, completely prevented the development of NAFLD in *Pemt^−/−^* mice: hepatic lipid levels, as well as markers of endoplasmic reticulum stress, inflammation, and fibrosis, in fenofibrate-treated *Pemt^−/−^* mice were similar to those in *Pemt^+/+^* mice. Importantly, *Pemt^−/−^* mice were still protected against HFD-induced obesity and insulin resistance. Moreover, fenofibrate partially reversed hepatic steatosis and fibrosis in *Pemt^−/−^* mice when treatment was initiated after NAFLD had already been established. Increasing hepatic fatty acid oxidation can compensate for the lower VLDL-triacylglycerol secretion rate and prevent/reverse fatty liver disease in mice lacking PEMT.

Phosphatidylethanolamine *N*-methyltransferase (PEMT) is an important enzyme for phosphatidylcholine (PC) synthesis in liver (1). As in all nucleated cells, PC in hepatocytes can be synthesized via the CDP-choline pathway. Hepatocytes, however, are unique in that they have a quantitatively important secondary pathway for PC synthesis, mediated by PEMT. In this pathway, phosphatidylethanolamine (PE) is sequentially methylated to form PC, which contributes 30% of hepatic PC synthesis (2).

In recent years, we have demonstrated that hepatic PEMT plays a role in several aspects of the metabolic syndrome: when mice lacking PEMT were fed a high-fat diet (HFD), they were strikingly protected from obesity and insulin resistance (3). However, *Pemt^−/−^* mice developed severe nonalcoholic fatty liver disease (NAFLD), which was predominantly caused by inadequate secretion of VLDLs (3, 4). Other factors, such as diminished hepatic fatty acid oxidation or increased fatty acid flux to the liver from diet or white adipose tissue (5), could also partly contribute to the development of hepatic steatosis in *Pemt^−/−^* mice. NAFLD is a chronic liver disease, affecting 10–24% of the population. NAFLD encompasses a wide spectrum of pathological symptoms, progressing from steatosis via nonalcoholic steatohepatitis (NASH), to fibrosis, cirrhosis, and eventually liver failure (6, 7). In *Pemt^−/−^* mice fed a HFD, fatty liver disease progresses rapidly. Hepatic steatosis was already evident after 4 days of HFD feeding, with a 5-fold higher hepatic triacylglycerol (TG) level compared with *Pemt^+/+^* mice (8). Within 2 weeks of HFD feeding, livers from *Pemt^−/−^* mice showed increased inflammation and cellular ballooning, as well as elevated expression levels of macrophage markers and cytokines, indicating NASH development (8). NASH was exacerbated when *Pemt^−/−^* mice were fed the HFD for 10 weeks, and the mice developed hepatic fibrosis (3, 5).

Lipid homeostasis in the liver is a balance between uptake and synthesis, and secretion and catabolism. This balance is disturbed in *Pemt^−/−^* mice: the reduced capacity to secrete VLDL particles results in severe TG accumulation in the liver (3–5, 9). We investigated whether this balance could be restored by increasing the amount of fat removed from the liver through the stimulation of fatty acid oxidation. PPARα is a nuclear receptor that, upon activation, induces the expression of a multitude of genes involved in fatty acid oxidation (10). Fibrates are synthetic PPARα agonists that are clinically used to treat dyslipidemia (11), and we investigated whether fenofibrate could ameliorate NAFLD in *Pemt^−/−^* mice.

Recently, several reports demonstrated that the cholesterol absorption inhibitor ezetimibe reduces hepatic steatosis (12–14). Ezetimibe inhibits the Niemann-Pick C1-like 1 protein in enterocytes, thereby reducing intestinal cholesterol absorption. Ezetimibe improved NAFLD in rats fed a methionine- and choline-deficient diet (15), as well as in HFD-fed Zucker obese rats (16). Ezetimibe also reduced hepatic fat accumulation in mice with diet-induced steatosis (12–14), possibly due to a reduction in sterol regulatory element-binding protein 1c mRNA (12). In all these models, plasma lipids (both TG and cholesterol) were reduced after treatment with ezetimibe. Concomitant with cholesterol, the absorption of saturated fatty acids was inhibited through lower levels of fatty acid binding protein in the intestines of mice after treatment with ezetimibe (17). Reduced fat absorption could lead to reduced delivery of TGs to the liver, and hence ameliorate diet-induced hepatic steatosis. Therefore, independent from increasing fatty acid oxidation, we also assessed whether hepatic steatosis could be reduced in mice lacking PEMT, by reducing intestinal fatty acid absorption and/or hepatic sterol regulatory element-binding protein 1c through inhibition of the Niemann-Pick C1-like 1 protein by ezetimibe.

We report that treatment with fenofibrate prevented and partially reversed NAFLD in *Pemt^−/−^* mice, whereas treatment with ezetimibe did not lower hepatic lipid accumulation, inflammation, or fibrosis in these mice.

## MATERIALS AND METHODS

### Animals

All experimental procedures were approved by the University of Alberta’s Institutional Animal Care Committee in accordance with guidelines of the Canadian Council on Animal Care. Male *Pemt^+/+^* and *Pemt^−/−^* mice (backcrossed into C57Bl/6 for seven generations; four to eight animals per group), 8 weeks old at the start of the study, were fed a semisynthetic HFD (catalog no. F3282, Bio-Serv, Flemington, NJ; 60 kcal% fat from lard) or the HFD supplemented with either fenofibrate (0.2% w/w; Sigma-Aldrich, St. Louis, MO) or ezetimibe (0.008% w/w; mixed thoroughly into the food) ad libitum for 6 weeks. With an average daily food intake of 75 g/kg body weight, this provided an approximate intake of 150 mg/kg/day for fenofibrate and 6 mg/kg/day for ezetimibe. Body weight was monitored weekly during the experiment. Another set of male *Pemt^+/+^* and *Pemt^−/−^* mice were fed a HFD for 2 weeks, after which half of the animals received the HFD supplemented with fenofibrate (0.1% w/w; 75 mg/kg/day), while the other half continued the HFD for 4 more weeks. Animals were fasted for 12 h before collection of blood by cardiac puncture. Tissues were collected, snap-frozen in liquid nitrogen, and stored at −80°C until further analyses. Samples for histological evaluation were fixed in formalin and subjected to hematoxylin-eosin and Picro-Sirius red (PSR) staining.

### In vivo metabolic tests

VLDL-TG production rates were measured in vivo. After a 12 h fast, mice received an intraperitoneal injection of Poloxamer 407 (1 g/kg). Blood samples were collected by tail bleeding before and at 1, 2, and 4 h after the injection of Poloxamer 407, and TG concentrations were measured. VLDL-TG production rate was calculated from the slope of the line of TG concentration versus time. For the glucose tolerance test, mice were fasted for 12 h, after which they received glucose (2 g/kg body weight) by oral gavage. Blood glucose levels were measured by using a glucometer (Accu-Chek, Indianapolis, IN) immediately before and at indicated times after administration. Total fat mass was determined by using a Minispec whole-body composition analyzer (Minispec LF90 II; Bruker, Hamilton, Canada).

### Analytical procedures

Plasma ketone bodies, total cholesterol, and free cholesterol were quantified by using commercially available kits from Wako Chemicals GmbH (Neuss, Germany). Hepatic PC, PE, and TG, as well as plasma TG and alanine aminotransferase (ALT), were quantified as described (5). For immunoblotting, livers were homogenized in buffer (100 mM Tris–HCl, 150 mM NaCl, 1 mM EDTA, 1 mM DTT, and 0.1 mM PMSF, pH 7.4) containing a protease inhibitor cocktail. Proteins were transferred to a polyvinylidene difluoride membrane, and the membrane was probed with primary antibodies against CCAAT/-enhancer-binding protein homologous protein (CHOP) (catalog no. 2895, Cell Signaling, Beverly MA), 78 kDa glucose-regulated protein (GRP78) (Cell Signaling, catalog no. 3183), apoB (catalog no. AB742, Chemicon, Billerica, MA), and calnexin (catalog no. ADI-SPA-865, Enzo, Farmingdale, NY). Immunoreactive proteins were detected by using the enhanced chemiluminescence system (GE Healthcare, Piscataway, NJ) according to the manufacturer’s instructions. Proteins were visualized by an enhanced chemiluminescence system (Amersham Biosciences, Piscataway, NJ) and quantified by using G:Box (Syngene, Cambridge, UK) software. RNA isolation, cDNA synthesis, and real-time quantitative PCR were performed as described (3). mRNA levels were normalized to cyclophilin or 18S mRNA. Fibrillar collagen in liver was visualized by PSR staining (5 μm sections, formalin-fixed livers) and fluorescence microscopy (18).

### Statistical analysis

Data were analyzed with GraphPad Prism software (GraphPad, La Jolla, CA). All values are means ± SEM. Data were tested for normal distribution and log-transformed when required. To compare groups, a two-way ANOVA was performed, followed by Fisher’s least significant difference (LSD) post hoc test. Statistical analyses of the tolerance tests were performed by using a two-way ANOVA for repeated measures. In addition, areas under the curves were calculated and analyzed by using a two-way ANOVA followed by Fisher’s LSD post hoc test. Level of significance of differences was *P* < 0.05.

## RESULTS

### Ezetimibe did not improve NAFLD in mice lacking PEMT

**Fig. 1.**
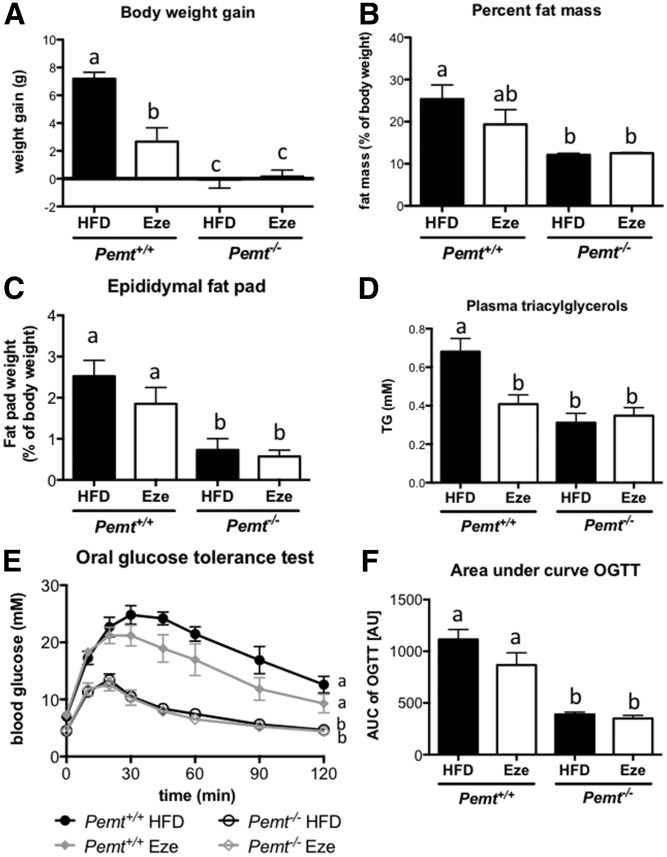
Ezetimibe reduced body weight gain in *Pemt^+/+^* mice, but did not decrease glucose tolerance. A: Body weight gain was measured in *Pemt^+/+^* and *Pemt^−/−^* mice after 6 weeks of feeding the HFD. B: Body composition was measured, and percent fat mass was calculated. C: Epididymal fat pads were collected and weighed. D: Plasma concentrations of TGs in *Pemt^+/+^* and *Pemt^−/−^* mice fed the HFD or HFD + ezetimibe. Glucose tolerance test (E) and area under the curve (F) in *Pemt^+/+^* and *Pemt^−/−^* mice fed the HFD or HFD + ezetimibe. Values are means ± SEM (*n* = 5 or 6 per group). The curves of the glucose tolerance test were compared by using two-way ANOVA for repeated measures. Values that do not share a letter are significantly different (*P* < 0.05). AU, arbitrary units; AUC, area under the curve; Eze, ezetimibe; OGTT, oral glucose tolerance test.

**Fig. 2.**
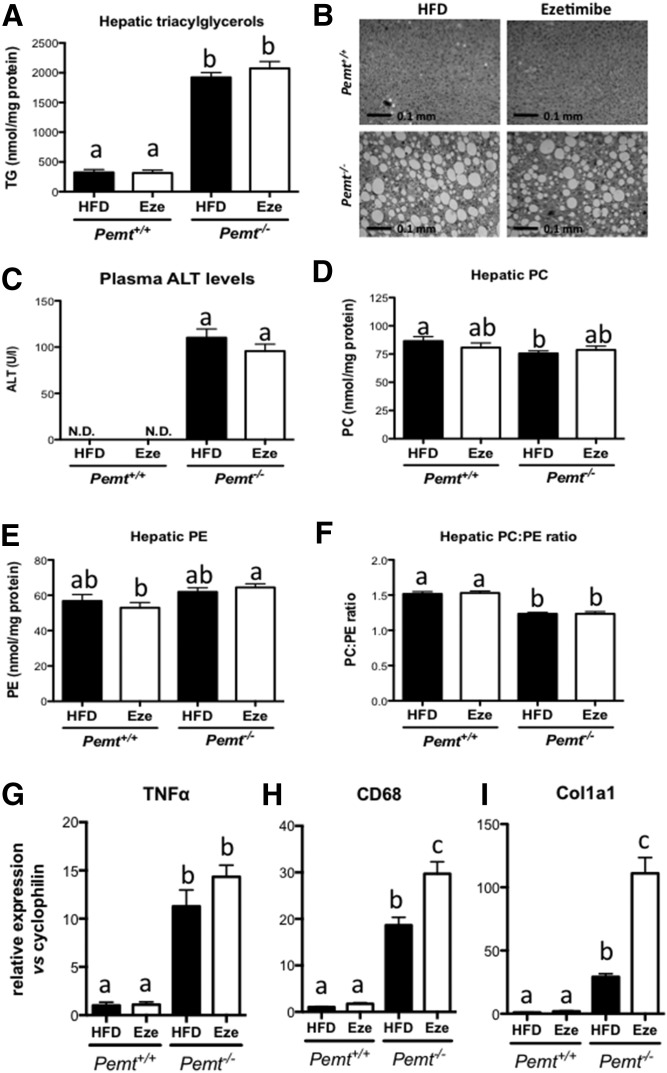
Ezetimibe did not improve NAFLD in *Pemt^−/−^* mice. A: Hepatic TG in *Pemt^+/+^* and *Pemt^−/−^* mice fed the HFD or HFD + ezetimibe. B: Representative pictures of hematoxylin and eosin staining of livers from untreated and treated *Pemt^−/−^* mice. C: Plasma levels of ALT as a marker of liver integrity. N.D., nondetectable. D–F: PC, PE, and PC:PE ratio in livers from *Pemt^+/+^* and *Pemt^−/−^* mice fed the HFD or HFD + ezetimibe. mRNA levels of genes involved in inflammation (G and H) and fibrosis (I) normalized to cyclophilin mRNA in livers from *Pemt^+/+^* and *Pemt^−/−^* mice fed the HFD or HFD + ezetimibe. Values are expressed relative to *Pemt^+/+^* fed HFD. Values are means ± SEM (*n* = 5 or 6 per group). Values that do not share a letter are significantly different (*P* < 0.05). Eze, ezetimibe.

Recent reports have indicated that treatment of mice with ezetimibe can prevent or reduce diet-induced hepatic steatosis (12, 14, 19). Therefore, we treated *Pemt^+/+^* and *Pemt^−/−^* mice with ezetimibe to determine whether it prevented hepatic steatosis in *Pemt^−/−^* mice. After 6 weeks of HFD feeding, *Pemt^−/−^* mice did not gain weight, whereas *Pemt^+/+^* mice gained 7.2 ± 0.5 g. Ezetimibe reduced weight gain of *Pemt^+/+^* mice by 63% (to 2.7 ± 1.0 g), which was partly due to reduced lipid mass (**Fig. 1A–C**). Concomitantly, *Pemt^−/−^* mice were more tolerant to an oral bolus of glucose than were *Pemt^+/+^* mice, and ezetimibe had no effect on glucose tolerance in *Pemt^−/−^* mice. In *Pemt^+/+^* mice, the reduction in weight gain on ezetimibe seemed to be accompanied by a slightly improved glucose tolerance; however, this did not reach statistical significance (Fig. 1E, F). Interestingly, plasma TG levels were reduced when *Pemt^+/+^* mice were treated with ezetimibe (Fig. 1D). Plasma TG in *Pemt^−/−^* mice was already low and was not altered by ezetimibe. Importantly, treatment of *Pemt^−/−^* mice with ezetimibe did not prevent or reduce the development of NAFLD. Hepatic TG levels were 6-fold higher in *Pemt^−/−^* mice compared with *Pemt^+/+^* mice, and treatment with ezetimibe did not reduce these levels (**Fig. 2A**). Histological evaluation confirmed excessive lipid accumulation in *Pemt^−/−^* mice, which was not ameliorated by ezetimibe treatment (Fig. 2B). Plasma ALT levels were elevated in *Pemt^−/−^* compared with *Pemt^+/+^* mice, and ezetimibe did not lower these levels (Fig. 2C). Hepatic PC was only slightly reduced in HFD-fed *Pemt^−/−^* mice compared with *Pemt^+/+^* mice, due to a compensatory upregulation of the CDP-choline pathway for PC biosynthesis (20) and/or a reduction of PC catabolism/secretion (5, 9). Nevertheless, the PC:PE ratio, which is often related to hepatic steatosis (21, 22), was lower in *Pemt^−/−^* mice (Fig. 2F). Ezetimibe did not affect hepatic PC or PE concentrations, nor did it change the PC:PE molar ratio in either genotype (Fig. 2D–F). Moreover, mRNA levels of markers for hepatic inflammation (*Tnfα* and *Cd68)* and fibrosis (*Col1a1*) were markedly elevated in *Pemt^−/−^* mice compared with *Pemt^+/+^* mice (Fig. 2G–I). Treatment with ezetimibe did not affect *Tnfα* mRNA and even increased *Cd68* mRNA in *Pemt^−/−^* mice (Fig. 2G, H). Similarly, mRNA levels for *Col1a1*, a marker for hepatic fibrosis, was 4-fold higher in *Pemt^−/−^* mice treated with ezetimibe (Fig. 2I). Thus, treatment with ezetimibe reduced weight gain and plasma TG levels in *Pemt^+/+^* mice, but worsened mRNA markers for NAFLD in mice lacking PEMT.

### Treatment of *Pemt^+/+^* mice with fenofibrate prevented diet-induced obesity and insulin resistance

**Fig. 3.**
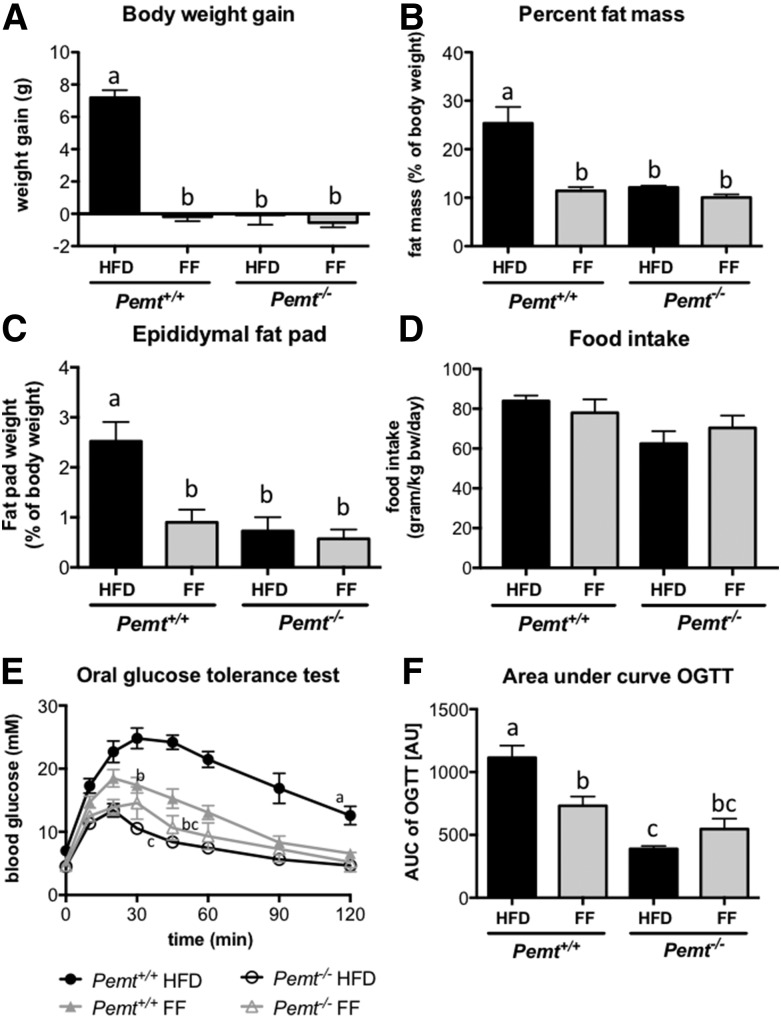
Fenofibrate reduced body weight gain and improved glucose tolerance in *Pemt^+/+^* mice. A: Body weight gain was measured in *Pemt^+/+^* and *Pemt^−/−^* mice after 6 weeks of feeding the HFD or HFD + fenofibrate. B: Body composition was measured, and percent fat mass was calculated. C: Epididymal fat pads were collected and weighed. D: Food intake was measured in *Pemt^+/+^* and *Pemt^−/−^* mice fed the HFD or HFD + fenofibrate. Glucose tolerance test (E) and area under the curve (F) in *Pemt^+/+^* and *Pemt^−/−^* mice fed the HFD or HFD + fenofibrate. Values are means ± SEM (*n* = 6–8 per group). The curves of the glucose tolerance test were compared by using two-way ANOVA for repeated measures. Values that do not share a letter are significantly different (*P* < 0.05). AU, arbitrary units; AUC, area under the curve; FF, fenofibrate; OGTT, oral glucose tolerance test.

We next investigated the impact of PPARα activation on the metabolic phenotype of *Pemt^−/−^* mice. When *Pemt^+/+^* mice were fed a HFD for 6 weeks, they developed obesity and insulin resistance, which did not occur in mice lacking PEMT (**Fig. 3**). Consistent with previous reports (23, 24), treatment of *Pemt^+/+^* mice with fenofibrate prevented diet-induced obesity (Fig. 3A). The reduction in body weight gain was mainly attributable to reduced fat mass in the *Pemt^−/−^* mice, as shown in Fig. 3B, C. Importantly, consistent with previous reports (3, 25, 26), food intake was not affected by either genotype or treatment (Fig. 3D). Associated with the reduced body weight and adiposity, glucose tolerance was also improved in *Pemt^+/+^* mice treated with fenofibrate (Fig. 3E, F). Besides the reduction in adiposity, improved glucose tolerance could also be reflecting changes in liver and brown adipose tissue. In brown adipose tissue, mRNA of hexokinase 2 was increased in *Pemt^+/+^* mice after fenofibrate treatment (supplemental Fig. S1A), which could suggest an increase in glucose uptake, flux through glucose-6-phosphate, and possibly glycolysis. Additionally, fenofibrate treatment increased Pgc1α mRNA in *Pemt^+/+^* mice, suggesting increased mitochondrial biogenesis. However, none of the measured genes involved in fatty acid oxidation in brown adipose tissue were affected by fenofibrate (supplemental Fig. S1A). As previously reported (25), fenofibrate strongly reduced glucokinase mRNA in liver. Moreover, hepatic gluconeogenesis seems to be reduced in *Pemt^+/+^* after treatment with fenofibrate (supplemental Fig. S1B). Mice lacking PEMT were already protected against diet-induced obesity and glucose intolerance, and fenofibrate did not have an additive effect on these parameters (Fig. 3), nor did it change mRNA levels of genes involved in glucose metabolism in liver or brown adipose tissue in *Pemt^−/−^* mice (supplemental Fig. S1A, B).

### Fenofibrate prevented steatohepatitis in *Pemt^−/−^* mice

**Fig. 4.**
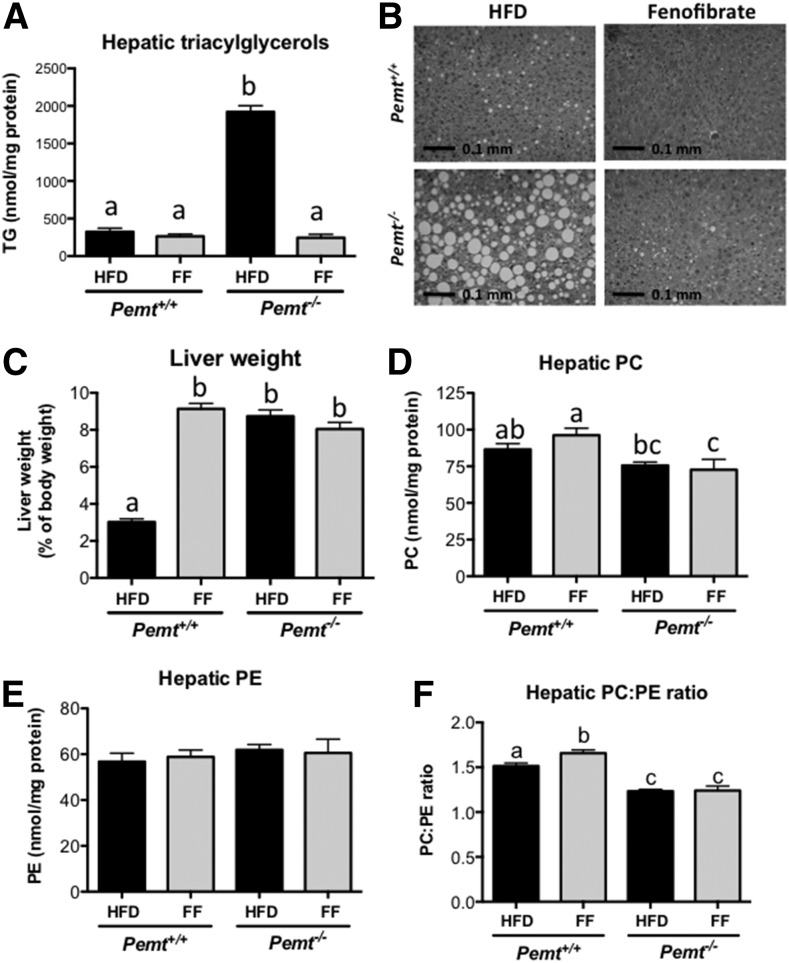
Fenofibrate improved hepatic steatosis in *Pemt^−/−^* mice. A: Hepatic TG was measured in *Pemt^+/+^* and *Pemt^−/−^* mice after 6 weeks of feeding the HFD or HFD + fenofibrate. B: Representative pictures of hematoxylin and eosin staining of livers from untreated and treated *Pemt^−/−^* mice. C: Liver weights of *Pemt^+/+^* and *Pemt^−/−^* mice fed the HFD or HFD + fenofibrate. Hepatic PC (D) and PE (E) and PC:PE ratio (F) were measured in *Pemt^+/+^* and *Pemt^−/−^* mice after 6 weeks of feeding the HFD or HFD + fenofibrate. Values are means ± SEM (*n* = 6–8 per group). Values that do not share a letter are significantly different (*P* < 0.05). FF, fenofibrate.

*Pemt^−/−^* mice develop severe fatty liver disease, including hepatic inflammation and fibrosis (3, 5). We hypothesized that hepatic steatosis might be prevented by stimulating hepatic fatty acid oxidation through PPARα activation. Indeed, hepatic TG accumulation in *Pemt^−/−^* mice was completely prevented by treatment of the mice with fenofibrate (**Fig. 4A**), and hepatic TG concentrations in treated *Pemt^−/−^* mice were similar to those in untreated *Pemt^+/+^* mice. Histological evaluation confirmed that the massive accumulation of lipid droplets in livers of *Pemt^−/−^* mice fed the HFD was entirely prevented when the mice were treated with fenofibrate (Fig. 4B). However, activation of PPARα with fenofibrate did not prevent the increased liver weight in *Pemt^−/−^* mice. As previously reported (27–29), fenofibrate caused hepatomegaly due to peroxisome proliferation (Fig. 4C). Fenofibrate did not change hepatic phospholipids levels, but slightly, yet significantly, increased the PC:PE ratio in *Pemt^+/+^* mice only (Fig. 4D–F).

**Fig. 5.**
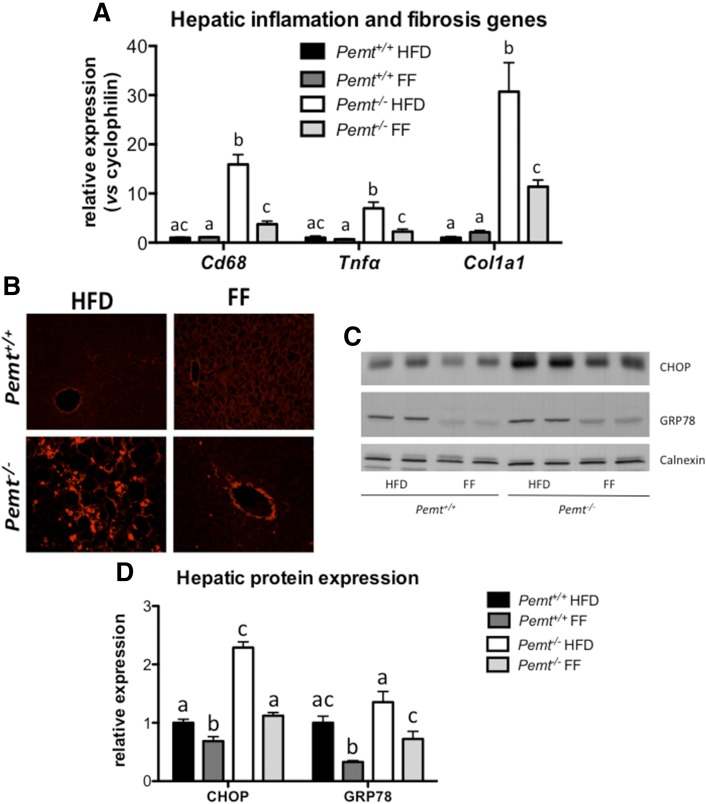
Fenofibrate reduced hepatic inflammation, fibrosis, and ER stress. A: mRNA levels of genes, normalized to cyclophilin mRNA, involved in inflammation and fibrosis in livers from *Pemt^+/+^* and *Pemt^−/−^* mice fed the HFD or HFD + fenofibrate. Values are expressed relative to *Pemt^+/+^* fed HFD. B: Histological assessment of hepatic fibrosis using PSR. C: Immunoblot of proteins involved in ER stress (CHOP and GRP78). D: Quantification of (B) relative to the amount of the loading control, calnexin. Values are means ± SEM (*n* = 6 per group for A; 3 per group for B; and 4 per group for C and D). Values that do not share a letter are significantly different (*P* < 0.05). FF, fenofibrate.

Besides preventing hepatic TG accumulation in *Pemt^−/−^* mice, fenofibrate treatment also prevented hepatic inflammation and fibrosis, as indicated by a reduction of mRNA levels of *Cd68* and *Tnfα*, both markers for inflammation, and *Col1a1*, a marker for fibrosis (**Fig. 5A**). In addition, histological analysis using PSR staining showed a clear reduction of fibrillar collagen in *Pemt^−/−^* livers after treatment with fenofibrate (Fig. 5B). Moreover, protein levels of CHOP [a marker of endoplasmic reticulum (ER) stress] were 2.5-fold elevated in *Pemt^−/−^* mice compared with *Pemt^+/+^* mice, and fenofibrate treatment normalized CHOP protein (Fig. 5C, D). Similarly, fenofibrate lowered the protein levels of GRP78, indicating that fenofibrate prevented ER stress in *Pemt^−/−^* livers.

**Fig. 6.**
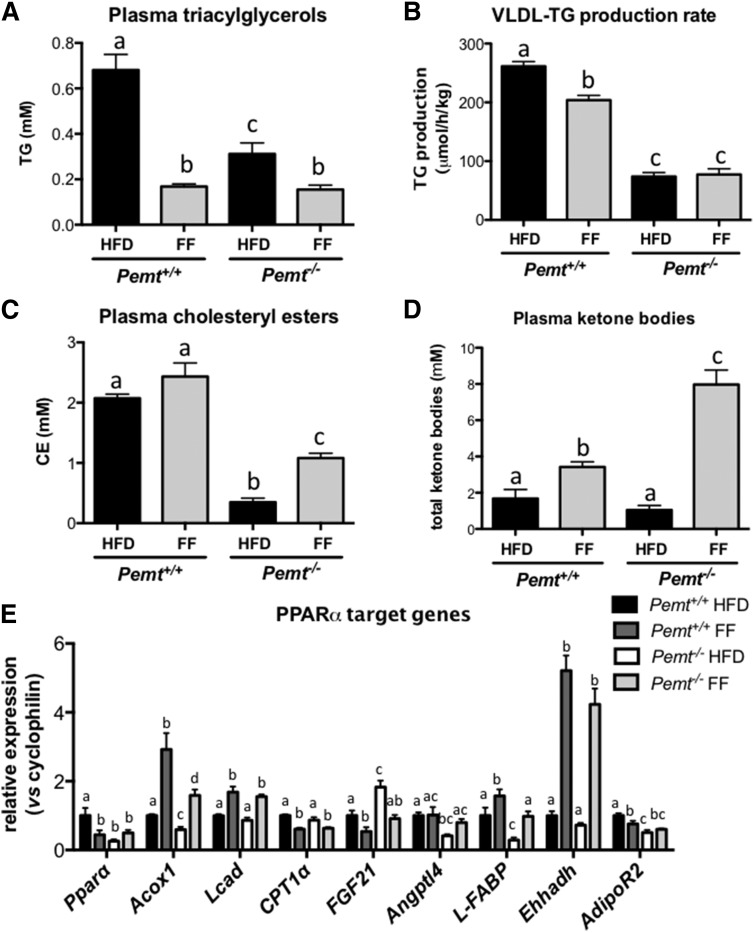
Fenofibrate stimulated fatty acid oxidation in both *Pemt* genotypes and reduced VLDL-TG secretion in *Pemt^+/+^* mice, but not in *Pemt^−/−^* mice. A: Plasma TG was measured in *Pemt^+/+^* and *Pemt^−/−^* mice after 6 weeks of feeding the HFD or HFD + fenofibrate. B: VLDL-TG production rate was calculated from the slope of plasma TG versus time curve after Poloxamer 407 injection in 12 h fasted mice. C: Plasma cholesteryl ester concentration was measured in *Pemt^+/+^* and *Pemt^−/−^* mice after 6 weeks of feeding the HFD or HFD + fenofibrate. D: Plasma ketone body concentrations in *Pemt^+/+^* and *Pemt^−/−^* mice fed the HFD or HFD + fenofibrate. E: mRNA levels of PPARα target genes normalized to cyclophilin mRNA in livers from *Pemt^+/+^* and *Pemt^−/−^* mice fed the HFD or HFD + fenofibrate. Values are expressed relative to *Pemt^+/+^* fed HFD. Values are means ± SEM (*n* = 6–8 per group). Values that do not share a letter are significantly different (*P* < 0.05). CE, cholesteryl ester; FF, fenofibrate.

TG accumulates in the livers of *Pemt^−/−^* mice mainly because the secretion of VLDL particles is impaired due to reduced rates of PC biosynthesis. Fasting plasma TG was lower in *Pemt^−/−^* mice compared with *Pemt^+/+^* mice (**Fig. 6A**), in agreement with reduced hepatic VLDL-TG secretion. Fenofibrate lowered fasting plasma TG in both genotypes, although to a greater extent in *Pemt^+/+^* mice. Fenofibrate also reduced the rate of VLDL-TG secretion in *Pemt^+/+^* mice by 20%, whereas it did not reduce VLDL secretion in *Pemt^−/−^* mice in which VLDL-TG secretion was already strongly diminished (Fig. 6B). Thus, it seems that increased TG secretion does not contribute to the prevention of hepatic steatosis in fenofibrate-treated *Pemt^−/−^* mice. Similarly, fenofibrate did not seem to reduced hepatic de novo lipogenesis: fenofibrate slightly, but significantly, increased hepatic mRNA levels of *Srebp1c* in *Pemt^+/+^* mice, but this did not translate into increased expression of the *Srebp1c* target genes *Acc* and *Fas* (supplemental Fig. S1C). In *Pemt^−/−^* mice, fenofibrate did not change *Srebp1c* mRNA levels, nor were the mRNAs for *Acc* and *Fas* levels different upon treatment. Thus, changes in lipogenesis did not appear to contribute to the prevention of hepatic steatosis in *Pemt^−/−^* mice. Interestingly, fenofibrate treatment increased plasma total and free cholesterol in both *Pemt^+/+^* and *Pemt^−/−^* mice (supplemental Fig. S2) and plasma cholesteryl esters in *Pemt^−/−^* mice only (Fig. 6C), which is likely due to its well-established effects on increasing HDL levels (reviewed in refs. 30–32).

To confirm that fenofibrate treatment indeed activated PPARα and thereby increased hepatic fatty acid oxidation, we measured plasma total ketone bodies (acetoacetone and 3-hydroxybutyrate). Plasma ketone bodies were 2-fold increased upon fenofibrate treatment of *Pemt^+/+^* mice, whereas the levels were 7.6-fold increased in *Pemt^−/−^* mice (Fig. 6D). Moreover, mRNA levels of *Acox*, *Lcad*, and *Ehhadh*, all involved in fatty acid oxidation, were increased after treatment with fenofibrate (Fig. 6E). In addition, the PPARα target gene *L-Fabp*, important for intracellular transport of fatty acids, was induced upon fenofibrate treatment. To our surprise, several other genes regulated by PPARα were not affected by fenofibrate treatment (Fig. 6E). Interestingly, mRNA levels of *Pparα* were lower in *Pemt^−/−^* compared with *Pemt^+/+^* mice. After fenofibrate treatment, *Pparα* mRNA was not different between genotypes. Thus, activation of PPARα completely prevented NAFLD in mice lacking PEMT, likely due to increased hepatic fatty acid oxidation.

### Fenofibrate partially reversed NAFLD in *Pemt^−/−^* mice

**Fig. 7.**
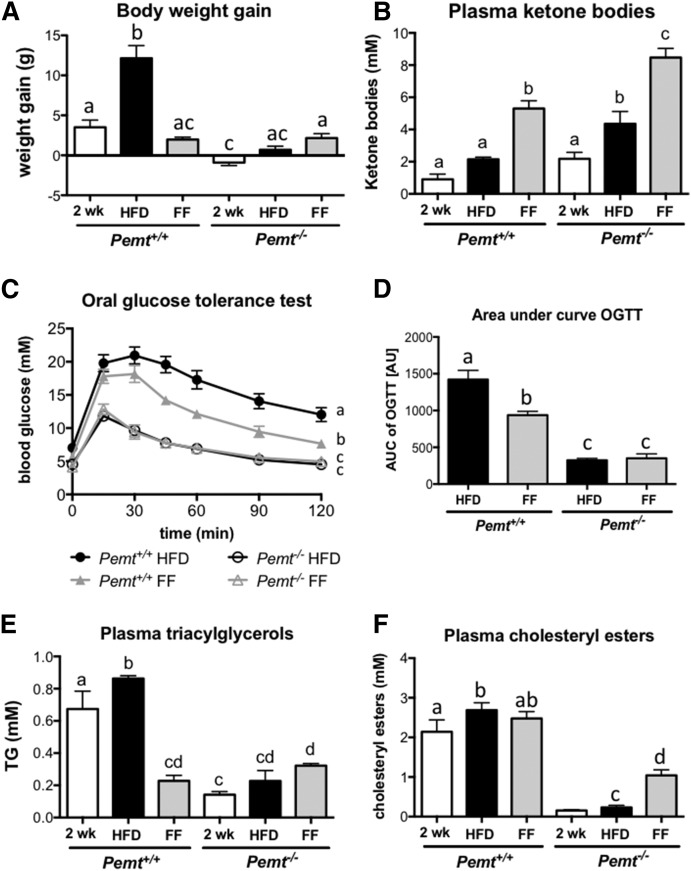
Fenofibrate increased plasma ketone bodies in *Pemt^−/−^* and *Pemt^+/+^* mice and improved glucose tolerance in *Pemt^+/+^* mice only. A: Body weight gain was measured in *Pemt^+/+^* and *Pemt^−/−^* mice fed the HFD for 2 or 6 weeks or for 2 weeks followed by 4 weeks of HFD supplemented with 0.1% fenofibrate. B: Plasma ketone body concentration in the same mice. Glucose tolerance test (C) and area under the curve (D) in *Pemt^+/+^* and *Pemt^−/−^* mice fed the HFD (black lines) or the HFD supplemented with 0.1% fenofibrate in the last 4 weeks of the study. Values are means ± SEM (*n* = 4–8 per group). The curves of the glucose tolerance test were compared by using two-way ANOVA for repeated measures. Plasma TGs (E) and cholesteryl esters (F) in *Pemt^+/+^* and *Pemt^−/−^* mice fed the HFD for 2 or 6 weeks or for 2 weeks followed by 4 weeks of the HFD supplemented with 0.1% fenofibrate. Values that do not share a letter are significantly different (*P* < 0.05). AU, arbitrary units; AUC, area under the curve; FF, fenofibrate; OGTT, oral glucose tolerance test.

Because treatment of *Pemt^−/−^* mice with fenofibrate completely protected the mice from developing steatohepatitis, we determined whether this treatment also reversed fatty liver disease after it had been established. To test this idea, we fed the mice a HFD for 2 weeks, which is a sufficient period for the *Pemt^−/−^* mice to develop NASH (8). At this time, mice were euthanized, kept on the HFD, or switched to a HFD containing 0.1% fenofibrate for 4 more weeks. *Pemt^+/+^* mice gained 3.5 g of body weight after 2 weeks of HFD feeding and continued to gain weight when HFD feeding was continued for 4 more weeks (12 g of body weight gain after 6 weeks on HFD; **Fig. 7A**). When fenofibrate was added to the diet in the last 4 weeks of feeding, further weight gain was prevented. *Pemt^−/−^* mice did not gain weight upon HFD feeding, and fenofibrate did not alter body weight in these mice. In line with reduced body weight, fenofibrate improved glucose tolerance in *Pemt^+/+^* mice, but had no effect in *Pemt^−/−^* mice (Fig. 7C, D). Importantly, fenofibrate-treated *Pemt^+/+^* mice did not become as tolerant to glucose as the *Pemt^−/−^* mice, even though body weight gain was similar (Fig. 7A, C, D). This suggests that the improved glucose tolerance in mice lacking PEMT is independent of weight gain. As seen above, fenofibrate increased plasma ketone bodies in both genotypes (Fig. 7B). Treatment reduced plasma TG in *Pemt^+/+^* mice, but not in *Pemt^−/−^* mice where levels were already low (Fig. 7E). Plasma cholesteryl esters were increased upon fenofibrate treatment in *Pemt^−/−^* mice only, likely due to an increase in HDL levels (Fig. 7F).

**Fig. 8.**
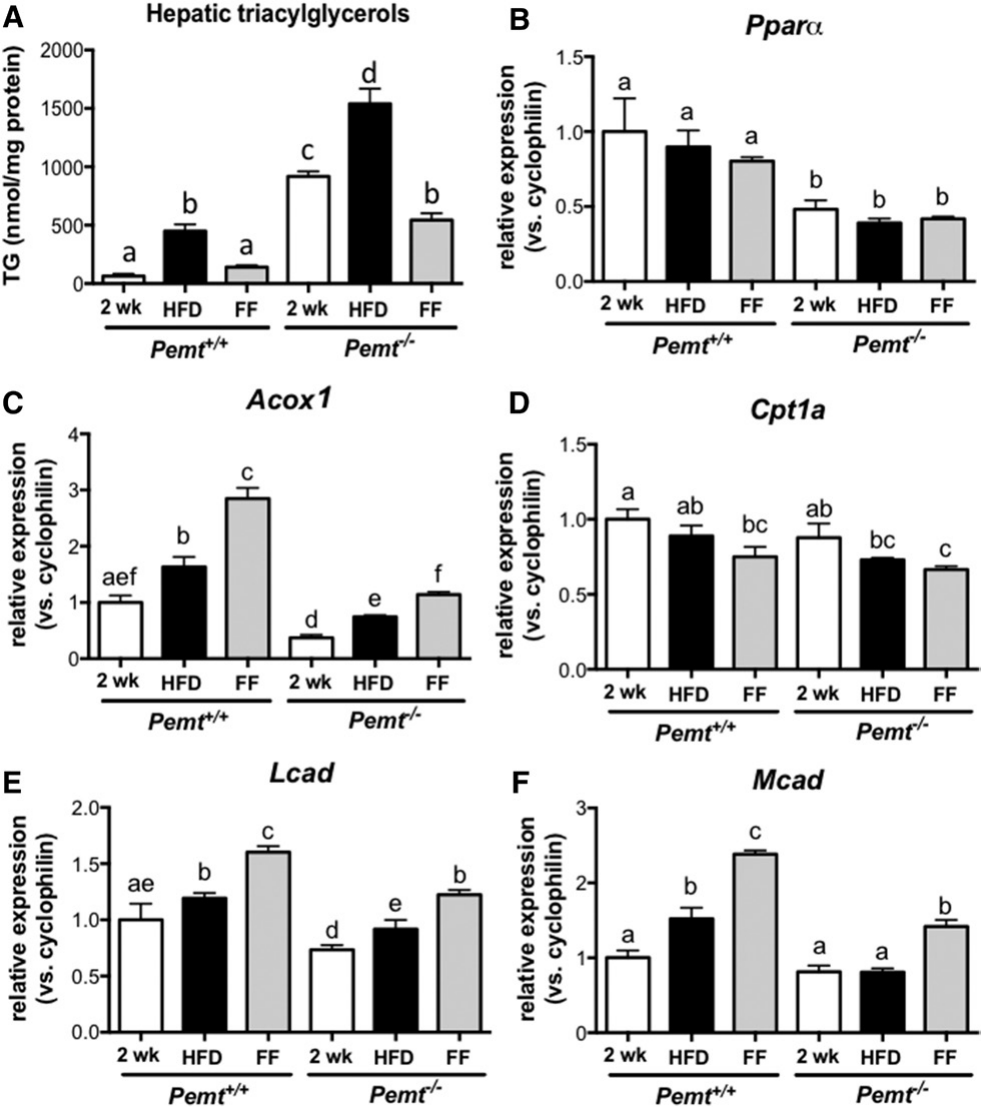
Fenofibrate reversed HFD-induced hepatic steatosis in *Pemt^−/−^* mice by increasing expression of genes involved in fatty acid oxidation. A: Hepatic TG levels in *Pemt^+/+^* and *Pemt^−/−^* mice fed the HFD for 2 or 6 weeks or for 2 weeks followed by 4 weeks of the HFD supplemented with 0.1% fenofibrate. B–F: Hepatic mRNA levels, normalized to cyclophilin mRNA, of genes involved in fatty acid oxidation in livers from *Pemt^+/+^* and *Pemt^−/−^* mice fed the HFD for 2 or 6 weeks or for 2 weeks followed by 4 weeks of the HFD supplemented with 0.1% fenofibrate. Values are expressed relative to *Pemt^+/+^* fed HFD for 2 weeks. Values are means ± SEM (*n* = 4–6 per group). Values that do not share a letter are significantly different (*P* < 0.05). FF, fenofibrate.

After 2 weeks of HFD feeding, TG accumulated in livers of *Pemt^−/−^* mice, which was exacerbated when HFD feeding was continued for 4 weeks. When the mice were switched to a diet containing 0.1% fenofibrate, hepatic TG levels did not increase and were even 35% lower compared with the levels after 2 weeks of HFD feeding (**Fig. 8A**). This finding indicates that fenofibrate treatment not only prevented the progression of, but also reversed, hepatic steatosis in *Pemt^−/−^* mice. We observed a similar pattern for *Pemt^+/+^* mice: hepatic TG accumulated during the 6 weeks of HFD feeding, and the progressive accumulation of TG stopped when the mice received fenofibrate during the last 4 weeks of the study. In *Pemt^+/+^* mice, however, hepatic TG levels after fenofibrate treatment were not lower than those after 2 weeks of HFD feeding (Fig. 8A). mRNA levels for *Acox*, *Lcad*, and *Mcad* were increased after fenofibrate treatment in both genotypes, suggesting that fatty acid oxidation was increased, which could result in reduced hepatic steatosis. Interestingly, in the same cohort of animals, the mRNAs for both *Pparα* and *Cpt1a* were not increased upon fenofibrate treatment (Fig. 8B–F).

**Fig. 9.**
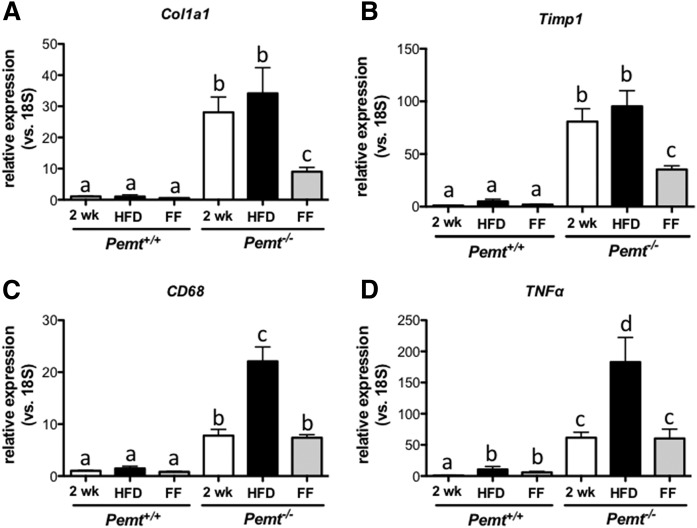
Fenofibrate reversed expression of markers of hepatic fibrosis in *Pemt^−/−^* mice. mRNA levels, normalized to 18S mRNA, of genes involved in hepatic fibrosis (A, B) and inflammation (C, D) in livers from *Pemt^+/+^* and *Pemt^−/−^* mice fed the HFD for 2 or 6 weeks or for 2 weeks followed by 4 weeks of the HFD supplemented with 0.1% fenofibrate. Values are expressed relative to *Pemt^+/+^* fed HFD for 2 weeks. Values are means ± SEM (*n* = 4–6 per group). Values that do not share a letter are significantly different (*P* < 0.05). FF, fenofibrate.

The mRNA markers for hepatic fibrosis (*Col1a1* and *Timp1* mRNA; **Fig. 9A, B**) showed a pattern similar to that of hepatic TG. The high expression levels of these mRNAs in HFD-fed *Pemt^−/−^* mice (after both 2 and 6 weeks) were reduced by ∼60% after treatment with fenofibrate, indicating regression of hepatic fibrosis by fenofibrate in *Pemt^−/−^* mice. Hepatic inflammation, as indicated by mRNA levels of *Cd68* and *Tnfα*, was elevated in *Pemt^−/−^* mice compared with *Pemt^+/+^* mice after 2 weeks of HFD feeding and further increased in *Pemt^−/−^* mice when HFD feeding continued for 4 more weeks. However, when fenofibrate was added to the diet in the last 4 weeks of the study, mRNA levels of *Cd68* and *Tnfα* did not further increase (Fig. 9C, D). Importantly, expression of *Cd68* and *Tnfα* after fenofibrate treatment was not lower than after 2 weeks of HFD, indicating that fenofibrate prevented the progression of, but did not reverse, hepatic inflammation in mice lacking PEMT.

## DISCUSSION

PEMT-deficient mice are protected from HFD-induced obesity and insulin resistance, but develop severe NAFLD. We have shown that treatment of *Pemt^−/−^* mice with fenofibrate, but not with ezetimibe, prevents and partially reverses NAFLD.

### Ezetimbe and NAFLD

Ezetimibe has been reported to reduce diet-induced hepatic steatosis in mice (12, 14, 19); however, the mechanism by which a cholesterol absorption inhibitor lowers hepatic TG is still unknown. Unlike in humans and rats, hepatic expression levels of NPC1L1, the target of ezetimibe, are extremely low in mice (33). Therefore, it is most likely that any effect of ezetimibe on hepatic lipids would be secondary to effects that occur in the intestine, where NPC1L1 is abundantly expressed. Labonté et al. (17) have shown that, besides cholesterol, absorption of saturated fatty acids is also reduced by ezetimibe, which could eventually lead to a reduced delivery of fatty acids for uptake into the liver. We observed a reduction in body weight gain and plasma TG in *Pemt^+/+^* mice treated with ezetimibe, suggesting that intestinal fat absorption might have been reduced. However, this reduction in plasma TG did not result in lowering of hepatic TG levels. Similarly, we did not see an improvement in NAFLD in *Pemt^−/−^* mice when treated with ezetimibe. Moreover, ezetimibe treatment seemed to enhance NAFLD in *Pemt^−/−^* mice, because mRNA levels for inflammation and fibrosis markers were increased.

### Role of PPARα in mouse models of NAFLD

Unlike ezetimibe, fenofibrate was very efficient in preventing the development of NASH in *Pemt^−/−^* mice. Even though fenofibrate did not restore hepatic phospholipid levels and the subsequently impaired VLDL secretion, increasing lipid catabolism was sufficient to prevent steatosis and the associated inflammation and fibrosis. Histologically, the livers of treated *Pemt^−/−^* mice were indistinguishable from those of *Pemt^+/+^* mice. Importantly, fenofibrate also partially reversed NAFLD in *Pemt^−/−^* when treatment was started after the disease was already established.

PPARα plays a key role in intracellular fatty acid metabolism, and it is highly expressed in metabolically active tissues, such as muscle, brown adipose tissue, and liver (34). PPARα governs fatty acid transport and β-oxidation, thereby decreasing lipid storage as it controls the expression of numerous genes involved in β-oxidation (31, 35). During fasting, adipose-derived fatty acids are delivered to the liver to be oxidized. PPARα mediates the response to fasting, and its expression is increased to accommodate the increased demand for fatty acid oxidation during periods of fasting (10). Mice that are deficient in PPARα display increased accumulation of lipids in the liver upon fasting. Similarly, these mice are also susceptible to diet-induced NASH: although high-fat feeding does normally not lead to NASH, it does induce hepatic steatosis and inflammation in mice lacking PPARα (10, 36, 37). A HFD elevates the flux of fatty acids to the liver, and mice lacking PPARα are not able to accommodate the increased demand for oxidation. Conversely, treatment of mice fed a HFD with a PPARα agonist have reduced hepatic steatosis and inflammation, further highlighting the critical role for PPARα in hepatic fatty acid catabolism. A widely used murine model of NAFLD is mice fed a methionine- and choline-deficient diet, displaying liver features that are very similar to human NAFLD progression (38). Another model of NAFLD is Apo-E2 knock-in mice fed a Western-type diet that display the early stages of NASH (39). In both animal models, treatment with a PPARα agonist improved hepatic steatosis, inflammation, and, in the case of methionine/choline deficiency, fibrosis. In contrast, deficiency of PPARα in these animal models exacerbated those hallmarks of NAFLD (39–42). In PEMT-deficient mice, where hepatic lipids mainly accumulate due to inadequate secretion, PPARα activation improves all aspects of NAFLD. Presumably, the reduction in TG accumulation in fenofibrate-treated *Pemt^−/−^* mice is due to increased fatty acid oxidation. We were surprised to see that not all of the measured PPARα target genes were induced upon treatment. One possibility is that, under the conditions used in this study, only peroxisomal, but not mitochondrial, β-oxidation was stimulated. Nevertheless, this seemed to be sufficient to prevent hepatic lipid accumulation in *Pemt^−/−^* mice. Moreover, hepatic expression of *Pparα* and some of its target genes were reduced in HFD-fed *Pemt^−/−^* mice compared with *Pemt^+/+^* mice. Although we have not been able to measure the rate of hepatic fatty acid oxidation, the low levels of *Pparα* suggest lower rates of fatty acid oxidation, which could also contribute to the lipid accumulation in *Pemt^−/−^* livers.

Although a critical role for PPARα in fatty acid catabolism has been well established, several studies have reported increased hepatic steatosis upon PPARα activation (25, 26, 43–45). Fenofibrate induced hepatic de novo lipogenesis and fatty acid elongation by increasing expression of *Srebp1c*, resulting in increased lipid accumulation (25, 45). We did see an increase in *Srebp1c* mRNA expression in *Pemt^+/+^* mice after fenofibrate treatment, but there was no effect on expression of *Srebp1c* target genes, nor did it increase TG accumulation. One of the reasons for this discrepancy could be that the animals were fasted for 4–7 h in the studies mentioned above, whereas we fasted the animals for 12 h, after which de novo lipogenesis is very low. Besides, in our study the mice were fed a HFD, not a chow diet. When fed a HFD, the majority of fatty acids in the liver are derived from diet and/or white adipose tissue, whereas the (relative) contribution of lipogenesis is far greater when mice are fed a chow diet. In other studies (26, 43), fenofibrate was administered to severely obese mice. Activation of PPARα directly or indirectly stimulates lipolysis and secretion of fatty acids from white adipose tissue (43, 46), which are then taken up by the liver. Therefore, fenofibrate treatment in obese animals can greatly increase the flux of fatty acids to the liver, which could exceed the increase in fatty acid oxidation and thus lead to a net accumulation of lipids in the liver. One of the reasons why fenofibrate reduces hypertriglyceridemia is its ability to inhibit hepatic VLDL secretion. However, inhibition of VLDL secretion can contribute to increased steatosis. This would not be the case in *Pemt^−/−^* mice, because VLDL secretion is already strongly impaired with no further reduction by fenofibrate. Finally, high doses of fenofibrate used in some of the studies mentioned above (26, 44) can elicit hepatic toxicity and cause mitochondrial dysfunction (47, 48), possibly leading to increased hepatic steatosis.

Remarkably, fenofibrate strongly reduced expression of markers for hepatic fibrosis in *Pemt^−/−^* mice when treatment was started after NAFLD was established, indicating a regression of hepatic fibrosis. Inflammation, conversely, was not reduced, but fenofibrate prevented further progression of hepatic inflammation. Because PPARα is not expressed in hepatic stellate cells (49), reversal of fibrosis is likely due to changes in hepatocytes. One important factor that can lead to hepatic fibrosis is an increase in oxidative stress. We have previously reported that livers from HFD-fed *Pemt^−/−^* mice exhibit high levels of oxidative stress (5). Hydrogen peroxide is the main reactive oxygen species that activates stellate cells to produce transforming growth factor-β and collagen (50, 51). PPARα directly stimulates catalase expression, which catalyzes the degradation of hydrogen peroxide and is thus very important in protecting cells from oxidative damage (52). Therefore, improvement of hepatic fibrosis could be due to a reduction in reactive oxygen species-induced activation of hepatic stellate cells. Other factors can also mediate the development of hepatic fibrosis, such as hepatocyte apoptosis, inflammation (release of chemokines and cytokines from Kupffer cells), and microRNAs [reviewed in (53, 54)]. PPARα has antiinflammatory properties (55, 56) and could thus ameliorate fibrosis through inhibition of inflammation. Further research is needed to determine whether PPARα can also affect the other mediators of hepatic fibrosis.

### Clinical use of fibrates to treat NAFLD

Fibrates are clinically used to treat mixed dyslipidemia (11). To date, however, only a few clinical pilot studies have been performed to determine the efficacy of PPARα agonists for NAFLD. A small clinical trial in which 16 NAFLD patients were treated with fenofibrate for 48 weeks showed a decrease in the proportion of patients with elevated liver enzymes and a reduction in hepatocyte ballooning, but no improvement in hepatic steatosis (57). Treatment of steatotic liver donors with bezafibrate for 2–8 weeks in combination with diet and exercise showed a decrease in macrovesicular steatosis; however, no placebo control subjects were included in the study (19). Another study showed a reduction in liver enzymes after 4 weeks of treatment with gemfibrozil (58); because no histology was performed in this study, steatosis could not be evaluated. Furthermore, a study in which 16 NAFLD patients were treated with clofibrate for 12 months showed no reduction in liver enzymes or improvement in histology (59). However, plasma TG levels were not reduced in these subjects, rendering efficacy of treatment questionable in this trial. No clear conclusion can be drawn from these clinical studies due to their limitations. Consequently, large randomized studies need to be performed with novel, more potent PPARα activators to determine efficacy for improving NAFLD.

Although these clinical trials have not shown an overall clear benefit of fenofibrate on NAFLD, it could be a valuable therapeutic in a subset of NAFLD patients with a similar etiology as seen with PEMT deficiency, such as NAFLD patients with impaired VLDL secretion (i.e., abetalipoproteinemia), impaired phospholipid metabolism, or impaired methylation capacity. More than 98 SNPs were identified in the human *PEMT* gene (60), of which some have been shown to have functional or physiological significance. For example, subjects with a SNP (−744 G-C) in the *PEMT* promoter were susceptible to liver malfunction when fed a diet low in choline (61). Another SNP (V175M) results in a partial loss of activity (36% reduced activity when tested in rat hepatoma cells) (62). This SNP was reported to be more prevalent in subjects with confirmed NAFLD than in control subjects (62). However, there was no association between this polymorphism and hepatic TG content in the Dallas Heart Study (63). This suggests that reduced PEMT activity is not sufficient to cause NAFLD, but could make subjects more susceptible to developing NAFLD when other risk factors, such as diabetes, low dietary choline intake, or excess calorie intake, come into play. In these subjects, fenofibrate or other, more potent PPARα activators or modulators could be an effective therapeutic for reducing fatty liver disease.

In conclusion, we have shown in our studies that enhancing hepatic fatty acid oxidation in PEMT-deficient mice results in a metabolically desirable animal model that is lean, highly sensitive to insulin, and exhibits no signs of steatohepatitis.

## Supplementary Material

Supplemental Data
