# Supplementary material for: Fenofibrate, but not ezetimibe, prevents fatty liver disease in mice lacking phosphatidylethanolamine N-methyltransferase
Source: J Lipid Res. 2017 Mar 29;58(4):656–67. doi: 10.1194/jlr.M070631 (PMC5392742; doi:10.1194/jlr.M070631)
Supplement: Supplemental Data [file supp_58_4_656__index.html]

Fenofibrate, but not ezetimibe, prevents fatty liver disease in mice lacking phosphatidylethanolamine N-methyltransferase — Fenofibrate, but not ezetimibe, prevents fatty liver disease in mice lacking phosphatidylethanolamine N-methyltransferase — Supplemental Data 

# Fenofibrate, but not ezetimibe, prevents fatty liver disease in mice lacking phosphatidylethanolamine *N*-methyltransferase

## Supplemental Data

- Supplemental data (.pdf, 220 KB) - Tables and figures
