# Supplementary material for: Fenofibrate, but not ezetimibe, prevents fatty liver disease in mice lacking phosphatidylethanolamine N-methyltransferase
Source: J Lipid Res. 2017 Mar 29;58(4):656–67. doi: 10.1194/jlr.M070631 (PMC5392742; doi:10.1194/jlr.M070631)
Supplement: Supplemental Data [file 10.1194_M070631_jlr.M070631-1.pdf]

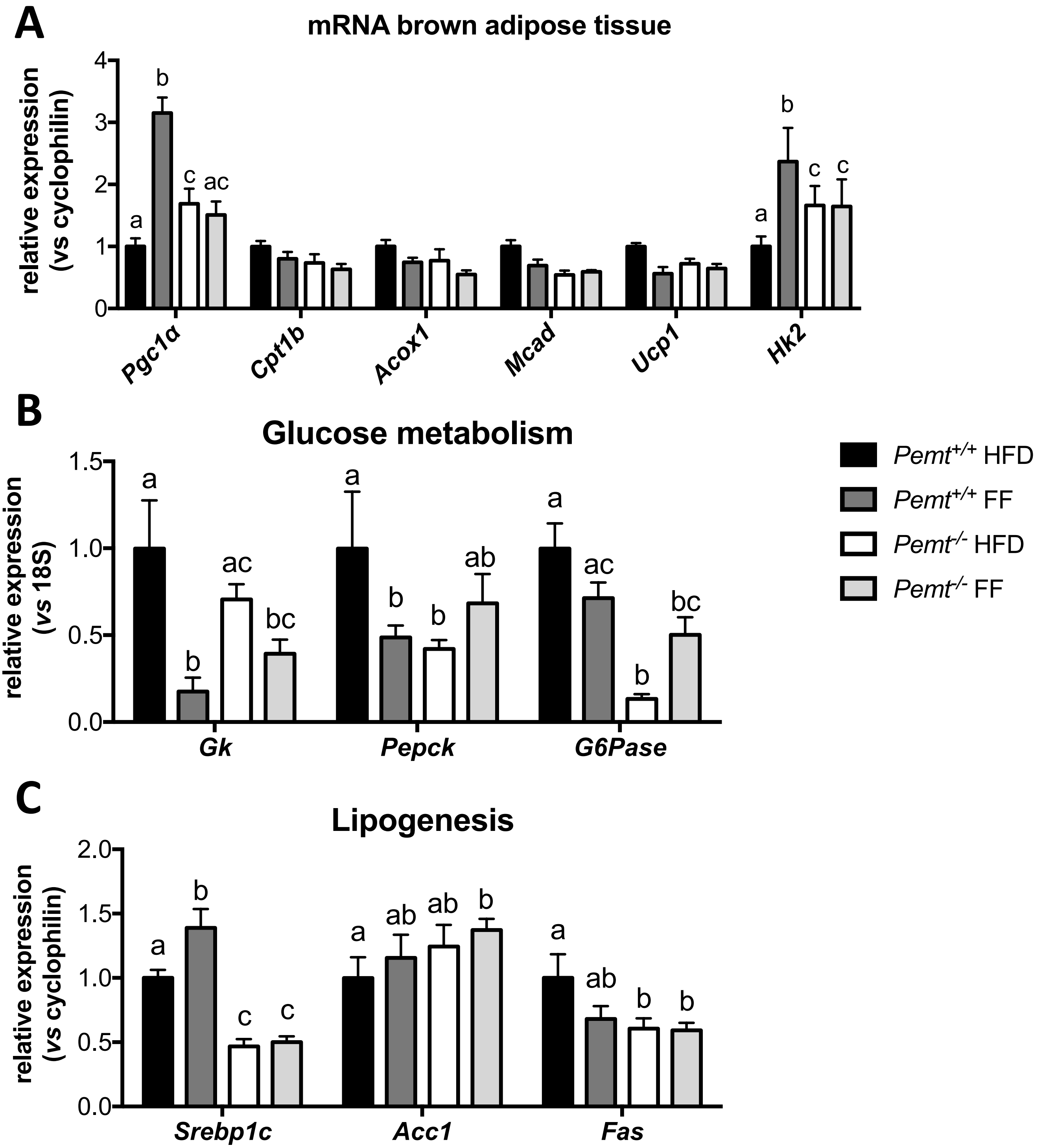

Supplemental Fig. S1. **Fenofibrate minimally affected expression of genes involved in glucose metabolism in brown adipose tissue and liver, and did not affect lipogenic gene expression.** mRNA levels of genes involved in energy metabolism in brown adipose tissue (A), in glucose metabolism in liver (B) and in de novo lipogenesis in liver (C) in *Pemt*$^{+/+}$ and *Pemt*$^{-/-}$ mice after 6 weeks of feeding the HFD or HFD + FF. Values are means ± SEM (n = 6-8 per group). ^{a,b,c}Values that do not share a letter are significantly different (P< 0.05).

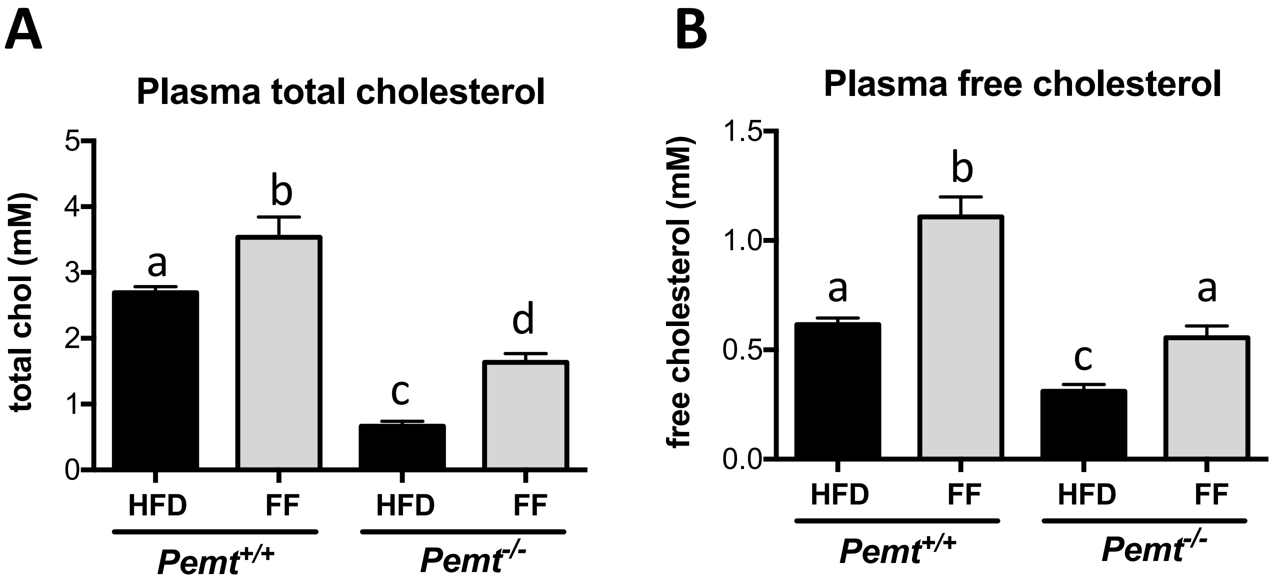

Supplemental Fig. S2. **Fenofibrate increased plasma total and free cholesterol in Pemt+/+ and Pemt-/- mice.** Plasma total cholesterol (A) and free cholesterol (B) concentrations were measured in *Pemt*$^{+/+}$ and *Pemt*$^{-/-}$ mice after 6 weeks of feeding the HFD or HFD + FF. Values are means ± SEM (n = 6-8 per group). [a,b,c]Values that do not share a letter are significantly different (P< 0.05).
